# A simulation study of sample size for multilevel logistic regression models

**DOI:** 10.1186/1471-2288-7-34

**Published:** 2007-07-16

**Authors:** Rahim Moineddin, Flora I Matheson, Richard H Glazier

**Affiliations:** 1 Centre for Research on Inner City Health, St. Michael's Hospital, Toronto, Canada; 2 Department of Public Health Sciences, University of Toronto, Toronto, Canada; 3 Department of Family and Community Medicine, University of Toronto, Canada; 4 Institute for Clinical Evaluative Sciences, Toronto, Canada

## Abstract

**Background:**

Many studies conducted in health and social sciences collect individual level data as outcome measures. Usually, such data have a hierarchical structure, with patients clustered within physicians, and physicians clustered within practices. Large survey data, including national surveys, have a hierarchical or clustered structure; respondents are naturally clustered in geographical units (e.g., health regions) and may be grouped into smaller units. Outcomes of interest in many fields not only reflect continuous measures, but also binary outcomes such as depression, presence or absence of a disease, and self-reported general health. In the framework of multilevel studies an important problem is calculating an adequate sample size that generates unbiased and accurate estimates.

**Methods:**

In this paper simulation studies are used to assess the effect of varying sample size at both the individual and group level on the accuracy of the estimates of the parameters and variance components of multilevel logistic regression models. In addition, the influence of prevalence of the outcome and the intra-class correlation coefficient (ICC) is examined.

**Results:**

The results show that the estimates of the fixed effect parameters are unbiased for 100 groups with group size of 50 or higher. The estimates of the variance covariance components are slightly biased even with 100 groups and group size of 50. The biases for both fixed and random effects are severe for group size of 5. The standard errors for fixed effect parameters are unbiased while for variance covariance components are underestimated. Results suggest that low prevalent events require larger sample sizes with at least a minimum of 100 groups and 50 individuals per group.

**Conclusion:**

We recommend using a minimum group size of 50 with at least 50 groups to produce valid estimates for multi-level logistic regression models. Group size should be adjusted under conditions where the prevalence of events is low such that the expected number of events in each group should be greater than one.

## Background

The idea that individual action is shaped by macro-level forces was evident in sociological theories of psychiatric illness and delinquency arising out of the Chicago School [1,2]. These theories suggest that while individual risk factors can affect individual health and delinquent behavior, so also can the structure of the social environment in which we live. It is only in the last 20 years that these theories could be truly tested, when statistical models were developed that allowed researchers to examine the additive and interactive effects of individual-level and contextual features that affect sociological outcomes at the individual level. In the last ten years the use of multilevel models has burgeoned in epidemiology. These models are highly appropriate in assessing how context affects individual-level health risks and outcomes [3].

Many kinds of data, including national surveys, have a hierarchical or clustered structure. For example, respondents in a complex large survey are naturally clustered in geographical units (e.g., health regions) and may be grouped into smaller units (e.g. census tracts). Over the last two decades, researchers have developed a class of statistical models designed for data with hierarchical structure. These models are variously known as mixed, hierarchical linear, random coefficient, and multilevel models. Hierarchical data routinely arise in many fields where multilevel models can be used as an extended version of the more traditional statistical techniques either to adjust for the dependency of the observations within clusters by using variables at higher levels or assessing the impact of higher level characteristics on the outcome after controlling for individual characteristics at the base level. An important feature of this class of models is the ability to estimate the cross-level interaction which provides a measure of the joint effect of a variable at the individual level in conjunction with a variable at the group level.

The robustness issue and the choice of sample size and power in multilevel modeling for continuous dependent variables has been studied by several authors [4-13]. Austin [14] used Monte Carlo simulation to assess the impact of misspecification of the distribution of random effects on estimation of and inference about both the fixed effects and the random effects in multilevel logistic regression models. He concluded that estimation and inference concerning the fixed effects were insensitive to misspecification of the distribution of the random effects, but estimation and inferences concerning the random effects were affected by model misspecification. Simulation studies indicate that a larger number of groups is more important than a larger number of individuals per group [4,5]. The overall conclusion from these studies is that the estimates of the regression coefficients are unbiased, but the standard errors and the variance components tend to be biased downward (underestimated) when the number of level 2 units is small (e.g. less than 30) [4,11].

Outcomes of interest in many fields do not only reflect continuous measures. Binary outcomes such as depression, presence or absence of a disease, and poor versus good self-reported general health are also of interest. Few studies have examined the accuracy of estimates, sample size or power analysis in binary multilevel regression [5,15]. Although Sastry et al. [15] calculate power and sample size in multilevel logistic regression models for their survey of children, families and communities in Los Angeles, they used a test of proportions between two comparison groups to calculate preliminary total sample size for a given baseline proportion and minimum detectable differences. After adjusting the calculated preliminary sample size for design effect, a total sample size of 3,250 was adopted. Finally based on simulation studies with total sample size of 3,250 and group sizes of 51, 66, 75, and 81 they decided to sample 65 groups (tracts) each of size 50.

We are unaware of any studies to date that have focused on these issues in multilevel logistic regression in a more comprehensive manner. In this paper simulation studies based on multilevel logistic regression models are used to assess the impact of varying sample size at both the individual and group level on the accuracy of the estimates of the parameters and their corresponding variance components.

## Methods

### Simulation models

We focus on the following multilevel logistic model with one explanatory variable at level 1 (individual level) and one explanatory variable at level 2 (group level):

logit(pij)=π0j+π1jxijπ0j=γ00+γ01zj+u0jπ1j=γ10+γ11zj+u1j
 MathType@MTEF@5@5@+=feaafiart1ev1aaatCvAUfKttLearuWrP9MDH5MBPbIqV92AaeXatLxBI9gBaebbnrfifHhDYfgasaacH8akY=wiFfYdH8Gipec8Eeeu0xXdbba9frFj0=OqFfea0dXdd9vqai=hGuQ8kuc9pgc9s8qqaq=dirpe0xb9q8qiLsFr0=vr0=vr0dc8meaabaqaciaacaGaaeqabaqabeGadaaakeaafaqaaeWabaaabaGaeeiBaWMaee4Ba8Maee4zaCMaeeyAaKMaeeiDaqNaeeikaGIaemiCaa3aaSbaaSqaaiabdMgaPjabdQgaQbqabaGccqGGPaqkcqGH9aqpiiGacqWFapaCdaWgaaWcbaGaeGimaaJaemOAaOgabeaakiabgUcaRiab=b8aWnaaBaaaleaacqaIXaqmcqWGQbGAaeqaaOGaemiEaG3aaSbaaSqaaiabdMgaPjabdQgaQbqabaaakeaacqWFapaCdaWgaaWcbaGaeGimaaJaemOAaOgabeaakiabg2da9iab=n7aNnaaBaaaleaacqaIWaamcqaIWaamaeqaaOGaey4kaSIae83SdC2aaSbaaSqaaiabicdaWiabigdaXaqabaGccqWG6bGEdaWgaaWcbaGaemOAaOgabeaakiabgUcaRiabdwha1naaBaaaleaacqaIWaamcqWGQbGAaeqaaaGcbaGae8hWda3aaSbaaSqaaiabigdaXiabdQgaQbqabaGccqGH9aqpcqWFZoWzdaWgaaWcbaGaeGymaeJaeGimaadabeaakiabgUcaRiab=n7aNnaaBaaaleaacqaIXaqmcqaIXaqmaeqaaOGaemOEaO3aaSbaaSqaaiabdQgaQbqabaGccqGHRaWkcqWG1bqDdaWgaaWcbaGaeGymaeJaemOAaOgabeaaaaaaaa@7352@

where [u0ju1j]=N([00],[σ02σ01σ01σ12])
 MathType@MTEF@5@5@+=feaafiart1ev1aaatCvAUfKttLearuWrP9MDH5MBPbIqV92AaeXatLxBI9gBaebbnrfifHhDYfgasaacH8akY=wiFfYdH8Gipec8Eeeu0xXdbba9frFj0=OqFfea0dXdd9vqai=hGuQ8kuc9pgc9s8qqaq=dirpe0xb9q8qiLsFr0=vr0=vr0dc8meaabaqaciaacaGaaeqabaqabeGadaaakeaadaWadaqaauaabeqaceaaaeaacqWG1bqDdaWgaaWcbaGaeGimaaJaemOAaOgabeaaaOqaaiabdwha1naaBaaaleaacqaIXaqmcqWGQbGAaeqaaaaaaOGaay5waiaaw2faaiabg2da9iabd6eaonaabmaabaWaamWaaeaafaqabeGabaaabaGaeGimaadabaGaeGimaadaaaGaay5waiaaw2faaiabcYcaSmaadmaabaqbaeqabiGaaaqaaGGaciab=n8aZnaaDaaaleaacqaIWaamaeaacqaIYaGmaaaakeaacqWFdpWCdaWgaaWcbaGaeGimaaJaeGymaedabeaaaOqaaiab=n8aZnaaBaaaleaacqaIWaamcqaIXaqmaeqaaaGcbaGae83Wdm3aa0baaSqaaiabigdaXaqaaiabikdaYaaaaaaakiaawUfacaGLDbaaaiaawIcacaGLPaaaaaa@5062@.

Here *P*_*ij *_is the probability that individual *i *in group *j *will experience the outcome, *x*_ij _is an explanatory variable on the respondent level, and *z*_*j *_is a group level explanatory variable. Model (1) can be written in the following single equation:

log*it*(*p*_*ij*_) = *γ*_00 _+ *γ*_10_*x*_*ij *_+ *γ*_01_*z *_*j *_+ *γ*_11_*x*_*ij*_*z *_*j *_+ *u*_0*j *_+ *u*_1*j*_*x*_*ij*_

In equation (2) the segment *γ*_00 _+ *γ*_10_*x*_*ij *_+ *γ*_01_*z*_*j *_+ *γ*_11_*x*_*ij *_*z *_*j *_is the fixed effect part and the segment *u*_0*j *_+ *u*_1*j*_*x *_*ij *_is the random part of the model. An important feature of equation (2) is the presence of a cross-level interaction term represented by *γ*_11_*z*_*j*_*x*_*ij *_in which the coefficient *γ*_11 _shows how *π*_1*j*_, the slope of equation (1), varies with *z*_*j*_, the group level variable.

The size of the intra-class correlation coefficient (ICC) may also affect the accuracy of the estimates [16]. The ICC for the logistic model is defined as ρ=σu2σu2+σe2
 MathType@MTEF@5@5@+=feaafiart1ev1aaatCvAUfKttLearuWrP9MDH5MBPbIqV92AaeXatLxBI9gBaebbnrfifHhDYfgasaacH8akY=wiFfYdH8Gipec8Eeeu0xXdbba9frFj0=OqFfea0dXdd9vqai=hGuQ8kuc9pgc9s8qqaq=dirpe0xb9q8qiLsFr0=vr0=vr0dc8meaabaqaciaacaGaaeqabaqabeGadaaakeaaiiGacqWFbpGCcqGH9aqpdaWcaaqaaiab=n8aZnaaDaaaleaacqWG1bqDaeaacqaIYaGmaaaakeaacqWFdpWCdaqhaaWcbaGaemyDauhabaGaeGOmaidaaOGaey4kaSIae83Wdm3aa0baaSqaaiabdwgaLbqaaiabikdaYaaaaaaaaa@3D4F@ where σe2=π23
 MathType@MTEF@5@5@+=feaafiart1ev1aaatCvAUfKttLearuWrP9MDH5MBPbIqV92AaeXatLxBI9gBaebbnrfifHhDYfgasaacH8akY=wiFfYdH8Gipec8Eeeu0xXdbba9frFj0=OqFfea0dXdd9vqai=hGuQ8kuc9pgc9s8qqaq=dirpe0xb9q8qiLsFr0=vr0=vr0dc8meaabaqaciaacaGaaeqabaqabeGadaaakeaaiiGacqWFdpWCdaqhaaWcbaGaemyzaugabaGaeGOmaidaaOGaeyypa0ZaaSaaaeaacqWFapaCdaahaaWcbeqaaiabikdaYaaaaOqaaiabiodaZaaaaaa@35DD@ and σu2
 MathType@MTEF@5@5@+=feaafiart1ev1aaatCvAUfKttLearuWrP9MDH5MBPbIqV92AaeXatLxBI9gBaebbnrfifHhDYfgasaacH8akY=wiFfYdH8Gipec8Eeeu0xXdbba9frFj0=OqFfea0dXdd9vqai=hGuQ8kuc9pgc9s8qqaq=dirpe0xb9q8qiLsFr0=vr0=vr0dc8meaabaqaciaacaGaaeqabaqabeGadaaakeaaiiGacqWFdpWCdaqhaaWcbaGaemyDauhabaGaeGOmaidaaaaa@3108@ is the variance of the random intercept in a fully unconditional multilevel logistic model log*it*(*p*_*ij*_) = *γ*_00 _+ *u*_0*j *_where *u*_0*j *_~ *N*(0, σu2
 MathType@MTEF@5@5@+=feaafiart1ev1aaatCvAUfKttLearuWrP9MDH5MBPbIqV92AaeXatLxBI9gBaebbnrfifHhDYfgasaacH8akY=wiFfYdH8Gipec8Eeeu0xXdbba9frFj0=OqFfea0dXdd9vqai=hGuQ8kuc9pgc9s8qqaq=dirpe0xb9q8qiLsFr0=vr0=vr0dc8meaabaqaciaacaGaaeqabaqabeGadaaakeaaiiGacqWFdpWCdaqhaaWcbaGaemyDauhabaGaeGOmaidaaaaa@3108@) [17].

The accuracy of the parameter estimates is quantified by the percentage relative bias [11]. Let θ^
 MathType@MTEF@5@5@+=feaafiart1ev1aaatCvAUfKttLearuWrP9MDH5MBPbIqV92AaeXatLxBI9gBaebbnrfifHhDYfgasaacH8akY=wiFfYdH8Gipec8Eeeu0xXdbba9frFj0=OqFfea0dXdd9vqai=hGuQ8kuc9pgc9s8qqaq=dirpe0xb9q8qiLsFr0=vr0=vr0dc8meaabaqaciaacaGaaeqabaqabeGadaaakeaaiiGacuWF4oqCgaqcaaaa@2E79@ stand for the estimate of the population parameter *θ*, then θ^−θθ×100
 MathType@MTEF@5@5@+=feaafiart1ev1aaatCvAUfKttLearuWrP9MDH5MBPbIqV92AaeXatLxBI9gBaebbnrfifHhDYfgasaacH8akY=wiFfYdH8Gipec8Eeeu0xXdbba9frFj0=OqFfea0dXdd9vqai=hGuQ8kuc9pgc9s8qqaq=dirpe0xb9q8qiLsFr0=vr0=vr0dc8meaabaqaciaacaGaaeqabaqabeGadaaakeaadaWcaaqaaGGaciqb=H7aXzaajaGaeyOeI0Iae8hUdehabaGae8hUdehaaiabgEna0kabigdaXiabicdaWiabicdaWaaa@37BB@ indicates the percentage relative bias for parameter *θ*. The accuracy of the standard error of the parameter estimate is assessed by analyzing the observed coverage of the 95% confidence interval created by using the asymptotic standard normal distribution [11].

Following the simulation conditions used by Maas and Hox [11] we set the following conditions for our simulation studies: (i) the number of individuals per group, *j*, *n*_*j*_, was set at 5, 30, and 50, (ii) the number of groups was set at 30, 50, and 100, and (iii) the variances of the random intercept were set at 0.13, 0.67, and 2.0, corresponding to intra-class correlation coefficients (ICC) of 0.04, 0.17, and 0.38, respectively. The individual and group explanatory variables *x*_*ij *_and *z*_*j *_are generated from the standard normal distribution. The group random components *u*_0*j *_and *u*_1*j *_are independent normal variables with mean zero and standard deviations *σ*_0 _and *σ*_1_where *σ*_1 _= 1 in all simulations and *σ*_0 _follows from the ICC and is set to 0.36, 0.82, and 1.42. We set the fixed effect parameters for all simulated models as: *γ*_00 _= -1.0, *γ*_01 _= 0.3, *γ*_10 _= 0.3, and *γ*_11 _= 0.3. To generate the outcome, a Bernoulli distribution with probability pij=exp⁡(π0j+π1jxij)1+exp⁡(π0j+π1jxij)
 MathType@MTEF@5@5@+=feaafiart1ev1aaatCvAUfKttLearuWrP9MDH5MBPbIqV92AaeXatLxBI9gBaebbnrfifHhDYfgasaacH8akY=wiFfYdH8Gipec8Eeeu0xXdbba9frFj0=OqFfea0dXdd9vqai=hGuQ8kuc9pgc9s8qqaq=dirpe0xb9q8qiLsFr0=vr0=vr0dc8meaabaqaciaacaGaaeqabaqabeGadaaakeaacqWGWbaCdaWgaaWcbaGaemyAaKMaemOAaOgabeaakiabg2da9maalaaabaGagiyzauMaeiiEaGNaeiiCaaNaeiikaGccciGae8hWda3aaSbaaSqaaiabicdaWiabdQgaQbqabaGccqGHRaWkcqWFapaCdaWgaaWcbaGaeGymaeJaemOAaOgabeaakiabdIha4naaBaaaleaacqWGPbqAcqWGQbGAaeqaaOGaeiykaKcabaGaeGymaeJaey4kaSIagiyzauMaeiiEaGNaeiiCaaNaeiikaGIae8hWda3aaSbaaSqaaiabicdaWiabdQgaQbqabaGccqGHRaWkcqWFapaCdaWgaaWcbaGaeGymaeJaemOAaOgabeaakiabdIha4naaBaaaleaacqWGPbqAcqWGQbGAaeqaaOGaeiykaKcaaaaa@5B3D@ is used. The overall prevalence of the outcome is close to 30 percent.

For practical purposes we generated 1000 data sets for each combination since a larger number of replications would have substantially increased processing time. The software SAS 9.1 (SAS Institute, North Carolina, US) was used for simulating observations and estimating the parameters. The SAS procedure NLMIXED with default options was used for estimation. This procedure only allows full maximum likelihood estimation. If convergence was not achieved the estimated parameters were not included in calculating summary statistics. We set initial values as the "true" values of each parameter. Distributions for random effects were normal, the optimization technique was Dual Quasi-Newton, and the integration method was Adaptive Gaussian Quadrature (AGQ). The number of quadrature points in AGQ was selected automatically. The absolute value for parameter convergence criterion was 10^-8 ^and the maximum number of iterations was 200.

## Results

### Convergence

The overall rate of model convergence varied from 56% to 100%. There were no negative variance estimates in converged models. Logistic regression was used to investigate the impact of ICC, number of groups and group size on the convergence. The rate of convergence (percent converged) significantly improved with either an increase in the number of groups or an increase in the group size. The overall rate of convergence for groups of sizes 5, 30, and 50 was 80.4%, 99.3, and 99.9%, respectively. For group of sizes 30, 50, and 100, the rate of convergence was 87.7%, 93.8%, and 98.1%. For the three ICC conditions of 0.04, 0.17, and 0.38 the rate of convergence was 89.2%, 94.5% and 95.9%. When we compared the samples that did and did not converge findings indicate no significant differences in prevalence (30.4% vs. 30.0%), mean and standard deviation of *z*_*j *_(0.01 and 1.01 versus 0.02 and 1.00), or mean and standard deviation of *x*_*ij *_(0.00 and 1.00 vs. 0.00 and 1.00). To further explore the non-convergent samples we examined 168 non-convergent simulated data sets with 30 groups and group size of 5. We first fitted a logistic model with random intercept only and then a logistic model with random slope only to each of these data sets. The estimated random intercepts and random slopes were classified as significant if the corresponding p-value was less that 0.05; otherwise each was classified as non-significant. Both the random intercept and random slope were statistically significant in only a small proportion of these data sets (2.4%). A closer investigation showed that when both random intercept and random slope were statistically significant either the random slope or the random intercept was severely underestimated. This suggests that non-convergence result from lack of sufficient variation in both the intercept and slope and further suggests that simplifying the model is appropriate; for example either a random intercept or a random slope is estimated, but not both.

### Distribution of parameter estimates

P-values and confidence intervals given by the NLMIXED procedure are based on asymptotic normality which may not be accurate for small sample sizes. The Shapiro-Wilk test calculates a *W *statistic that tests whether a random sample of size *n *comes from a normal distribution. The Shapiro-Wilk test for normality was used to test the normality of the distribution of fixed effect estimates for different combinations. Logistic regression was used to assess the effects of each factor on normality. The ICC was not associated with normality of the parameter estimates. The number of groups was associated with normality of the estimates for *γ*_10 _and *γ*_01 _group size was associated with normality of the estimates for *γ*_00_, *γ*_10_, and *γ*_11_. The majority of estimates from simulations with a group size of 5 were non-normal even with 100 groups. For simulations with a group size of 30 a few estimates were non-normal even with 50 groups. All estimates were normally distributed with 100 groups and group size of 50.

### Parameter estimates

In simulation studies of multilevel regression with continuous outcomes, Maas and Hox [11] found negligible bias for the fixed effect parameter estimates. They reported an average bias less than 0.05% for the fixed parameter estimates, intercept and the regression slopes. Our simulations show that the overall biases for the fixed effect parameters *γ*_00_, *γ*_01_, *γ*_10_, and *γ*_11 _were 0.6%, 2.6%, 1.4%, and 3.7% respectively (data not shown). The cross-level interaction parameter (*γ*_11_) had the largest overall bias.

Table 1 shows the percent relative bias and rate of convergence (percent converged) for different simulation conditions. For the fixed effect parameters, the largest biases (8.8%, 11.1%, 15.8%, and 13.3% for *γ*_00_, *γ*_01_, *γ*_10_, and *γ*_11_) were found under conditions where of the smallest variance for the random intercept (0.13), the smallest group size (5), and the lowest number of groups (30). When the size of the group was increased to 30 with 30 groups, the bias was reduced to less than 6%. These biases were reduced to less than 4% when the size of the group was 30 and the number of groups was 50. Even further reductions occurred (bias of 1% or less) when the size of the group was 30 and there were 100 groups.

**Table 1:** The effect of number of groups, group size, and ICC on the relative bias (θ^−θθ×100
 MathType@MTEF@5@5@+=feaafiart1ev1aaatCvAUfKttLearuWrP9MDH5MBPbIqV92AaeXatLxBI9gBaebbnrfifHhDYfgasaacH8akY=wiFfYdH8Gipec8Eeeu0xXdbba9frFj0=OqFfea0dXdd9vqai=hGuQ8kuc9pgc9s8qqaq=dirpe0xb9q8qiLsFr0=vr0=vr0dc8meaabaqaciaacaGaaeqabaqabeGadaaakeaadaWcaaqaaGGaciqb=H7aXzaajaGaeyOeI0Iae8hUdehabaGae8hUdehaaiabgEna0kabigdaXiabicdaWiabicdaWaaa@37BB@) of estimates.

Number of groups	Group size	ICC	% converged	*γ*_00_	*γ*_01_	*γ*_10_	*γ*_11_	*σ*_0_	*σ*_1_
30	5	0.04	56	8.77	11.12	15.85	13.26	174.04	55.49
		0.17	68	4.75	11.56	10.70	14.93	24.25	54.55
		0.38	76	3.94	12.22	5.91	14.89	15.82	54.02
	30	0.04	94	0.07	1.09	-1.93	3.57	-7.07	-6.81
		0.17	100	-0.08	3.70	-1.60	3.44	-5.89	-7.02
		0.38	100	-0.18	5.74	-1.72	5.31	-3.55	-6.92
	50	0.04	99	-0.39	0.69	-0.18	5.32	-8.47	-7.71
		0.17	100	-0.43	2.65	-1.70	5.74	-6.25	-7.16
		0.38	100	-0.39	4.62	-2.85	4.49	-5.05	-7.30
50	5	0.04	71	3.82	9.32	4.93	5.88	110.80	35.84
		0.17	86	1.44	8.40	2.34	4.73	11.95	25.55
		0.38	90	1.00	5.96	3.50	6.68	6.80	28.11
	30	0.04	99	0.09	0.21	2.01	2.72	-5.16	-2.60
		0.17	100	-0.32	0.28	2.06	3.69	-2.90	-2.55
		0.38	100	-0.37	-0.56	1.89	3.46	-3.24	-2.81
	50	0.04	100	-0.23	0.08	1.25	2.31	-6.18	-3.54
		0.17	100	-0.33	0.51	1.39	2.56	-3.76	-3.59
		0.38	100	-0.53	0.83	1.29	1.73	-3.19	-3.23
100	5	0.04	87	1.64	0.14	2.42	1.55	47.87	7.64
		0.17	98	0.47	-0.50	1.11	0.89	1.84	3.46
		0.38	98	0.95	0.25	2.25	0.41	2.23	8.82
	30	0.04	100	-0.02	-0.12	0.31	0.41	-5.13	-1.25
		0.17	100	-0.11	0.36	-0.06	0.96	-2.14	-1.17
		0.38	100	-0.21	1.04	-0.96	1.07	-2.06	-1.30
	50	0.04	100	0.03	0.35	-0.09	0.48	-3.28	-2.01
		0.17	100	-0.06	0.71	0.05	0.63	-2.12	-1.34
		0.38	100	-0.14	0.62	0.50	1.18	-1.73	-1.52

The estimates of the random intercept and random slope have larger biases compared to the fixed effect parameters. The overall biases (data not shown) for *σ*_0 _and *σ*_1_ were 6.9% and 5.0%. The bias for *σ*_1 _remained at the level of 5% for different values of *σ*_0_, however the estimates for *σ*_0 _had the largest bias (21.2%) for *σ*_0 _= 0.36.

The relative bias for the variance components was less than 4% when the size of the group was 50 and there were 100 groups. The variance-covariance parameter estimates are positively biased in all cases when the group size was 5 regardless of the number of groups (some exceeded 100%). The variance components were consistently underestimated when with a group size of 30 or more regardless of the number of groups. This problem of underestimation has been noted previously in simulation studies of multilevel models for continuous outcomes [11].

The overall relative bias for the random intercept was 21%, 0.5%, 0.1% for ICC 0.04, 0.17, 0.38, respectively. For the random slope, the overall relative biases for the three ICC conditions were not statistically different, ranging from 4% to 6%. There were no statistically significant differences in bias for the fixed effect parameters for any of the ICC conditions.

### Standard errors

We adopted the method used by Maas and Hox [11] to assess the accuracy of the standard errors. For each parameter in each simulated data set the 95% Wald confidence interval is established. For each parameter a non-coverage indicator variable is set to zero if the confidence interval contains the true value, otherwise if the true value lies outside the 95% confidence interval it is set to 1. The effect of number of groups, group size, and ICC on the non-coverage is presented in Tables 2 and 3, respectively. Logistic regression was used to assess the effect of the different simulated conditions on non-coverage.

**Table 2:** Non-coverage of the asymptotic 95% confidence interval by number of groups, group size, and ICC.

	Number of groups			p-value	Group size			p-value	ICC			p-value
Parameter	30	50	100		5	30	50		0.04	0.17	0.38	
*γ*_00_	0.053	0.058	0.049	0.036	0.046	0.053	0.059	0.0012	0.050	0.055	0.055	0.3507
*γ*_01_	0.057	0.056	0.055	0.865	0.047	0.059	0.060	0.0005	0.053	0.058	0.056	0.3760
*γ*_10_	0.059	0.055	0.051	0.058	0.043	0.060	0.059	0.0000	0.055	0.055	0.055	0.9948
*γ*_11_	0.060	0.058	0.049	0.004	0.044	0.059	0.061	0.0000	0.056	0.055	0.056	0.8946
*σ*_0_	0.106	0.090	0.089	0.000	0.097	0.094	0.093	0.7173	0.106	0.090	0.089	0.0003
*σ*_1_	0.095	0.077	0.070	0.000	0.057	0.085	0.094	0.0000	0.080	0.080	0.080	0.9820

**Table 3:** Effect of number of groups, group size, and ICC on the non-coverage of the asymptotic Wald 95% confidence interval.

Number of Groups	Group size	ICC	*γ*_00_	*γ*_01_	*λ*_10_	*γ*_11_	*σ*_0_	*σ*_1_
30	5	0.04	0.029	0.048	0.032	0.032	0.142	0.045
		0.17	0.049	0.046	0.040	0.028	0.090	0.041
		0.38	0.042	0.046	0.041	0.055	0.081	0.030
	30	0.04	0.061	0.051	0.070	0.068	0.095	0.111
		0.17	0.060	0.067	0.066	0.064	0.117	0.104
		0.38	0.053	0.061	0.066	0.062	0.107	0.111
	50	0.04	0.053	0.061	0.064	0.071	0.104	0.127
		0.17	0.062	0.063	0.063	0.063	0.108	0.116
		0.38	0.054	0.059	0.071	0.071	0.113	0.114
50	5	0.04	0.031	0.045	0.050	0.048	0.117	0.038
		0.17	0.051	0.041	0.040	0.044	0.070	0.056
		0.38	0.062	0.049	0.040	0.046	0.076	0.064
	30	0.04	0.050	0.067	0.059	0.064	0.091	0.092
		0.17	0.060	0.055	0.064	0.060	0.088	0.083
		0.38	0.062	0.060	0.059	0.057	0.094	0.076
	50	0.04	0.065	0.058	0.056	0.069	0.091	0.091
		0.17	0.065	0.060	0.053	0.070	0.102	0.091
		0.38	0.067	0.064	0.066	0.062	0.087	0.088
100	5	0.04	0.042	0.037	0.038	0.043	0.172	0.052
		0.17	0.046	0.058	0.055	0.047	0.070	0.078
		0.38	0.056	0.052	0.048	0.048	0.082	0.084
	30	0.04	0.045	0.046	0.057	0.055	0.085	0.061
		0.17	0.046	0.064	0.053	0.055	0.086	0.064
		0.38	0.039	0.058	0.049	0.050	0.087	0.066
	50	0.04	0.063	0.059	0.055	0.042	0.086	0.075
		0.17	0.053	0.063	0.052	0.050	0.079	0.067
		0.38	0.053	0.056	0.051	0.051	0.069	0.076

As shown in Table 2 the effect of number of groups on the standard errors of the fixed effect parameters is small with non-coverage rates ranging from 5% and 6%. The nominal non-coverage rate is 5%. The effect of number of groups on the standard errors of the variance component was larger than the nominal 5%, with non-coverage ranging from 7% to 11%. With 30 groups the non-coverage rate was 11% for the random intercept and 10% for the random slope. These non-coverage rates were reduced to 9% and 7% percent, respectively, for 100 groups. The extent of non-coverage implies that the standard error for the variance components is underestimated, a phenomenon reported by Maas and Hox [11] in their simulation studies of two-level linear regression models. The rate of non-coverage decreased as number of groups increased however, the non-convergence cannot be ignored.

The rates of non-coverage for the fixed effect parameters varied between 4 to 6% which is close to 5% nominal (Table 2). The effect of group size on the standard error of the estimates of the random intercept (close to 10%) was not significant; however the rate of non-coverage for the random slope increased as the group size increased. Table 2 shows that ICC had no effect on the non-coverage rates for the fixed effects or the random slope. Similar to findings for the number of groups and group size, the rate of non-coverage is close to 5% for the fixed effect parameters and over 5% for the random effect parameters. The rate of non-coverage for the random intercept decreased as ICC increased.

Table 3 shows the rates of non-coverage for each simulation condition. The minimum and maximum rates of non-coverage for the fixed effect parameters, *γ*_00_, *γ*_01_, *γ*_10_, and *γ*_11_, range from 3% and 7%. The rates of non-coverage for the variance-covariance components range from 7% and 17%. These findings indicate that the estimates of the standard errors are acceptable for the fixed effect parameters but not acceptable for the variance covariance components.

### Prevalence

The accuracy of the estimates of the parameters at the individual level depends on the prevalence of the outcome. To assess the relationship between the prevalence of the outcome and the sample size we repeated our simulations with prevalence rates of 0.10, 0.34, and 0.45. We set the parameters, *γ*_00_, *γ*_01_, *γ*_10_, and *γ*_11,_  at -3.0, 0.3, 0.3, and 0.3 for prevalence of 10%, at -1.0, 0.3, 0.3, and 0.3 for prevalence of 34% and -0.3, -0.3, -0.3, and -0.3 for prevalence of 45%. The variances of the random intercepts and random slopes were 1 for all simulations. Table 4 shows that for both fixed and random effect parameters the simulated data with 10% prevalence had the largest bias.

**Table 4:** Percent bias, non-coverage, and convergence rate for different prevalence of 0.10, 0.34, and 0.45.

	Bias			Non-coverage			% Converged		
Parameter	0.10	0.34	0.45	0.10	0.34	0.45	0.10	0.34	0.45
*γ*_00_	1.41	0.29	-0.37	0.055	0.056	0.054	77	86	87
*γ*_01_	3.78	2.94	0.20	0.054	0.057	0.062	77	86	87
*γ*_10_	1.12	0.44	0.61	0.053	0.054	0.052	77	86	87
*γ*_11_	4.35	3.63	-0.29	0.058	0.054	0.055	77	86	87
*σ*_0_	2.93	0.16	-0.18	0.107	0.088	0.085	76	86	86
*σ*_1_	8.40	4.41	3.35	0.091	0.081	0.084	76	86	86

The overall effect of prevalence on the non-coverage rates was not significant (data not shown). As shown in Table 4 the rate of non-coverage for all fixed effect parameter estimates ranged from 5 to 6%. The rate of non-coverage for the random intercept and random slope variance estimates ranged from 8 to 11%. This suggests that a larger sample size is necessary to minimize bias for low-prevalent outcomes. The largest bias was observed under conditions when the size of the group was 5 and the prevalence of the outcome was 10% (12% for fixed effect and 50% for random effect). Similarly, with 30 groups and a 10% prevalence the largest bias was 9% for the fixed effect parameters and 15% for the random slope. The rate of convergence was lowest with 10% prevalence.

## Discussion and conclusion

In this paper we investigated the impact of varying sample size at both the group and individual level on the accuracy of the parameter estimates and variance components using multilevel modeling for logistic regression. We also examined the effect of prevalence of the outcome on the accuracy of the estimates. The number of replications was restricted to 1000 due to extensive computer processing time.

Previous research has indicated that a sample of 50 groups and 30 units per group is sufficient to produce reliable parameter estimates for linear multilevel regression models [11]. Our findings suggest this may not be the case for logistic regression. Simulations presented in this paper suggest that the number of level two groups and the number of individuals in each group should be adjusted for prevalence of the outcome. Low prevalent events require a larger number of individuals per group.

We did not study the effect of different estimation procedures on the accuracy of the parameter estimates. However Rodriguez and Goldman [18,19] showed that the marginal quasi likelihood with first order Taylor expansion underestimates both the fixed effect and the variance-covariance components. The data set that formed the basis for these conclusions was extreme in the sense that the variance components were large and the sample size at the lowest level was quite small. In less extreme cases it appears that predictive quasi likelihood with second order Taylor expansion usually provides accurate estimates for both fixed and random parameters [5]. Simulations by Callens and Croux [20] compared penalized quasi-likelihood (PQL) with adaptive Gaussian quadrature (AGQ) and non-adaptive Gaussian quadrature (NGQ) and showed that PQL suffers from large bias but performs better in terms of mean-squared error (MSE) than standard versions of quadrature methods. They also showed that automatic selection of the number of quadrature points in AGQ (the default of the NLMIXED procedure) might be inadequate and lead to a loss in MSE. Thus, numerical results may change slightly depending on the statistical package, number of iterations, or algorithm used to estimate the parameters.

In multilevel analysis non-convergence can occur when estimating too many random components that are close to zero. Hox [5] suggests a solution to this problem which is to remove some random components, thereby simplifying the model. In our case non-convergence was a significant problem when group size was 5 and the number of groups was 30. There were no significant differences in the prevalence of the outcome or in the distribution of the explanatory variables among the converged versus non-converged samples. When the sample size is small there may not be sufficient variation to estimate a random effect, thus leading to non-convergence.

Simulation studies come with their own set of limitations. This said, our results are comparable with simulation results for multilevel regression models as reported by other researchers [11]. We focused on the impact of sample size at the individual and group level on the bias and accuracy of parameter estimates. We did not consider the impact of varying the distribution and variance of the individual and group level explanatory variables for practical reasons, specifically due to the large number of conditions that would have to be considered and which would result in extensive computer processing time. For a discussion of the impact of misspecification of the distribution of random effects on estimation of and inference about both the fixed effects and the random effects in multilevel logistic regression models see Austin [14].

Our results and recommendations are based on extensive simulation studies from data which are generated from normal distributions. Since the normal distribution assumption may be violated in real study applications we conducted further simulations with 100 replications for each model and relaxed the normal distribution assumption. This allowed us to compare convergence, coverage, and bias of the simulated non-normal models with the simulated normal model. Comparisons were done by each parameter, number of groups, and group size. The distributions for generating *z*_*j*_, *u*_0*j*_, *u*_1*j*_, and *x*_*ij *_for 8 simulated models are as follows: Model 1: N(0,1), N(0,1), N(0,1), N(0,1); Model 2: N(0,1), N(0,1), N(0,4), N(0,4); Model 3: N(0,4), N(0,4), N(0,4), N(0,4); Model 4: U(-0.5,0.5), N(0,1), N(0,1), U(-0.5,0.5); Model 5: U(-0.5,0.5), U(-2,2), U(-2,2), U(-0.5,0.5); Model 6: N(0,1), t(df = 3), t(df = 3), N(0,1); Model 7: t(df = 3), t(df = 3), t(df = 3), t(df = 3); Model 8: t(df = 5), t(df = 5), t(df = 5), t(df = 5) where N(a, b) stands for a normal distribution with mean a and variance b, U(a, b) represents uniform distribution over the interval (a, b), and t(df = k) stands for t-student distribution with k degrees of freedom. Table 5 shows the convergence, coverage and relative bias for above 8 models.

**Table 5:** Percent models converged, percent relative bias, and percent non-coverage (in brackets).

			Group Size = 5							Group Size = 30							Group Size = 50					
Model	Number of Groups	% converged	*γ*_00_	*γ*_01_	*γ*_10_	*γ*_11_	*σ*_0_	*σ*_1_	% converged	*γ*_00_	*γ*_01_	*γ*_10_	*γ*_11_	*σ*_0_	*σ*_1_	% converged	*γ*_00_	*γ*_01_	*γ*_10_	*γ*_11_	*σ*_0_	*σ*_1_
1	30	80	-3.9 (25)	12.3 (22)	-0.6 (23)	2.2 (22)	14.2 (29)	44.9 (25)	100	-1.9 (4)	-4.6 (6)	7.0 (5)	-5.4 (8)	-3.4 (11)	-9.7 (14)	100	-2.8 (5)	2.7 (6)	5.9 (2)	-9.0 (4)	-7.1 (15)	-9.8 (9)
2		84	0.3 (18)	18.0 (20)	8.0 (19)	-16.0 (20)	41.1 (31)	4.8 (25)	100	-1.0 (7)	1.4 (8)	9.7 (2)	-21.6 (6)	-1.7 (13)	-5.3 (9)	100	-1.8 (5)	0.9 (4)	6.0 (3)	-25.0 (7)	-5.6 (11)	-6.4 (11)
3		99	-2.3 (6)	0.7 (4)	-12.5 (2)	-11.0 (8)	5.8 (19)	0.8 (18)	100	-3.9 (6)	0.4 (5)	15.5 (1)	-7.7 (3)	-4.5 (17)	-5.9 (9)	100	-3.2 (7)	3.5 (3)	15.1 (4)	-8.4 (7)	-4.1 (12)	-4.3 (12)
4		41	12.9 (60)	8.3 (62)	70.9 (60)	-215 (61)	33.8 (64)	469 (64)	76	-0.2 (31)	-11.2 (30)	4.0 (26)	-29.0 (27)	0.3 (30)	9.7 (24)	94	0.61 (11)	-9.2 (10)	-1.4 (12)	35.1 (16)	0.3 (16)	-4.7 (14)
5		53	16.5 (50)	54.1 (49)	-7.6 (50)	-103 (47)	58.9 (50)	507 (52)	93	-0.9 (12)	28.3 (8)	7.1 (7)	43.0 (13)	3.2 (13)	0.2 (12)	97	0.7 (12)	-4.3 (6)	-9.0 (6)	23.4 (13)	-1.5 (9)	2.2 (10)
6		93	2.1 (9)	-9.6 (11)	23.9 (8)	15.3 (11)	-13.3 (27)	1.9 (20)	100	-3.2 (5)	-1.2 (5)	-4.1 (4)	-3.3 (8)	-15.3 (30)	-7.3 (24)	100	-3.3 (4)	-4.3 (4)	-5.0 (6)	-14.0 (6)	-15.0 (29)	-3.9 (28)
7		76	2.5 (5)	-4.8 (3)	27.1 (3)	12.9 (4)	-0.6 (19)	-11.5 (23)	99	-4.4 (13)	-10.7 (7)	0.0 (8)	-3.6 (7)	-18.9 (33)	-26.6 (39)	97	-4.6 (18)	-15.0 (7)	-0.7 (7)	-2.7 (10)	-15.5 (28)	-20.3 (35)
8		80	-1.0 (23)	-4.9 (25)	17.2 (23)	18.6 (24)	-1.5 (28)	-2.5 (27)	100	1.2 (7)	-2.4 (11)	-8.2 (11)	5.9 (12)	-15.1 (25)	-19.3 (26)	100	2.6 (6)	-0.7 (12)	-5.5 (10)	3.6 (10)	-7.1 (18)	-20.7 (32)
1	50	88	5.4 (14)	-4.3 (16)	-6.1 (13)	-4.3 (17)	19.2 (15)	23.5 (15)	100	3.0 (7)	4.7 (5)	-0.5 (9)	-4.2 (4)	2.1 (14)	-1.8 (10)	100	3.4 (9)	2.0 (8)	-0.3 (5)	-4.18 (7)	-3.9 (11)	-4.1 (6)
2		94	10.5 (10)	-0.8 (7)	-2.5 (10)	-5.9 (10)	22.4 (17)	15.3 (15)	100	5.0 (7)	6.5 (7)	-6.4 (8)	-7.8 (6)	-0.4 (8)	-0.2 (10)	100	3.3 (6)	4.8 (8)	-7.8 (8)	-10.0 (4)	-3.8 (10)	-3.6 (7)
3		99	11.9 (4)	14.7 (4)	-1.7 (8)	5.6 (5)	20.1 (10)	13.8 (14)	100	7.1 (6)	4.2 (7)	-6.5 (7)	-5.2 (7)	-2.9 (13)	-3.4 (5)	100	6.0 (7)	3.7 (6)	-8.4 (8)	-3.3 (4)	-2.8 (12)	-2.9 (9)
4		55	11.7 (54)	17.6 (50)	52.1 (52)	129 (53)	17.9 (54)	162 (58)	92	0.9 (14)	21.1 (11)	7.3 (17)	-36.1 (11)	-5.4 (18)	-6.3 (14)	100	0.9 (4)	32.0 (7)	-6.3 (8)	-14.6 (7)	-5.3 (9)	-3.5 (9)
5		58	5.2 (46)	-30.3 (49)	49.9 (46)	4.0 (47)	25.9 (53)	224 (52)	98	3.3 (8)	5.6 (7)	9.1 (9)	-34.3 (11)	7.1 (2)	-2.6 (7)	100	2.1 (5)	10.2 (7)	5.9 (9)	-48.3 (7)	4.3 (4)	-6.8 (9)
6		100	1.5 (3)	-4.0 (7)	-0.5 (4)	2.12 (3)	-15.0 (20)	-11.4 (22)	100	-4.1 (4)	-0.3 (8)	3.4 (4)	-4.7 (5)	-12.9 (35)	-16.9 (32)	100	-3.0 (6)	-1.1 (10)	1.1 (5)	-10.7 (6)	-6.2 (28)	-10.4 (30)
7		80	2.4 (4)	8.3 (6)	27.6 (2)	8.2 (2)	-3.7 (9)	-6.3 (20)	98	-1.4 (7)	-2.6 (9)	7.0 (6)	-2.4 (8)	-11.0 (22)	-22.5 (38)	95	-5.7 (7)	-4.0 (8)	8.1 (3)	0.0 (5)	-9.3 (25)	-20.8 (36)
8		98	-3.1 (4)	3.2 (4)	-11.1 (4)	5.4 (8)	-3.5 (11)	-3.5 (19)	100	-2.6 (8)	-0.8 (10)	-13.4 (6)	1.2 (3)	-9.0 (13)	-9.0 (17)	100	-3.3 (9)	0.7 (7)	-10.4 (7)	0.8 (3)	-10.1 (18)	-5.6 (18)
1	100	100	-0.5 (2)	-8.2 (7)	5.4 (3)	2.3 (4)	0.9 (4)	2.3 (10)	100	-2.4 (6)	0.8 (7)	2.6 (4)	-6.9 (4)	-2.6 (10)	-1.7 (4)	100	-2.4 (6)	-0.3 (13)	3.1 (3)	-6.0 (3)	-4.0 (11)	-2.8 (4)
2		100	-2.5 (6)	-1.8 (2)	1.4 (5)	-11.0 (5)	11.8 (6)	1.9 (9)	100	-1.9 (5)	-0.4 (12)	6.5 (2)	-11.9 (4)	-6.3 (9)	-1.6 (5)	100	-2.2 (7)	0.0 (15)	3.7 (0)	-8.4 (4)	-4.8 (8)	-3.6 (3)
3		100	-3.4 (4)	-0.9 (6)	-0.7 (1)	0.6 (5)	4.6 (12)	2.4 (9)	100	-5.6 (3)	-0.3 (9)	6.4 (4)	-5.9 (3)	-3.0 (8)	-2.4 (8)	100	-5.1 (7)	-2.1 (10)	6.5 (2)	-4.7 (5)	-3.7 (7)	-3.7 (6)
4		66	3.4 (39)	22.5 (38)	32.2 (38)	-8.4 (36)	1.7 (41)	137 (38)	100	-0.4 (4)	12.2 (7)	3.9 (3)	7.3 (5)	-0.7 (7)	-3.7 (10)	100	0.1 (7)	17.0 (9)	-7.9 (3)	-29.5 (8)	-1.3 (10)	-1.5 (10)
5		69	7.7 (40)	5.7 (34)	-12.6 (33)	-57.5 (34)	19.4 (38)	169 (36)	99	0.8 (7)	-11.5 (6)	-0.5 (8)	-20.1 (11)	6.2 (2)	0.4 (5)	100	0.9 (6)	-10.9 (3)	-4.6 (6)	-16.5 (9)	5.0 (1)	1.3 (4)
6		100	-4.0 (4)	4.1 (4)	2.7 (2)	17.2 (7)	-13.8 (16)	-20.9 (22)	100	-6.0 (11)	1.1 (5)	3.7 (3)	10.7 (10)	-14.6 (29)	-18.2 (34)	100	-5.8 (10)	-3.8 (4)	1.3 (4)	10.4 (7)	-9.7 (28)	-10.8 (32)
7		94	-3.0 (7)	1.6 (7)	16.1 (2)	6.2 (5)	-16.6 (22)	-26.0 (26)	98	-2.6 (10)	-1.6 (7)	12.2 (3)	0.4 (4)	-13.9 (33)	-22.5 (46)	94	-3.2 (11)	-0.6 (8)	8.8 (5)	-3.2 (7)	-13.6 (35)	-17.8 (37)
8		100	0.6 (7)	9.2 (4)	-10.4 (5)	6.4 (7)	-2.2 (9)	-10.2 (10)	100	1.9 (8)	4.0 (3)	0.3 (6)	-0.3 (4)	-4.5 (16)	-8.1 (17)	100	1.1 (4)	3.1 (5)	-2.0 (3)	2.3 (7)	-5.8 (20)	-6.4 (11)

Logistic regression was used to compare the rate of convergence of models 2 to 8 with model 1 (the model with 4 normal standard distributions). Models 3, 6, 7, and 8 were more likely to converge while models 4 and 5 were less likely to converge (p-value < 0.01) when group size was 5. When group size was greater than 5 there was no significant difference between the rates of convergence for all models.

The comparisons (Ttest) between the fixed effect parameter estimates of models 2–8 with model 1 did not show any significant differences. For models 6, 7, and 8 the random intercept and random slope were underestimated compare to model 1 (p < 0.01). This phenomenon was also observed and reported by Austin [14] for a logistic model with random intercept.

Fisher exact test was used to test the rate of coverage of models 2–8 with model 1 for each parameter, number of groups, and group size. There was no significant difference between the rates of coverage for the fixed effect parameters when group size was greater than 5 using groups of 50 or more. The rates of coverage for the random components of models 6 and 7 were significantly lower than the coverage rates of model 1 (p < 0.01). Austin [14] reported similar conclusions for a logistic model with random intercept.

Although the misspecification of random components significantly affected the estimates and standard errors of the random intercepts and random slopes when either group size or number of groups was small, the estimates and standard errors of all models were statistically the same as those estimates and standard errors for model 1 when the number of groups and group size was 50 or more; the exception being for a t-distribution with 3 degrees of freedom.

Despite the limitations of simulation studies [see for example 21] our findings can offer some suggestions for sample size selection in multilevel logistic regression. In practice a group size of 30 is often recommended in educational research and a group size of 5 is recommended in family and longitudinal research studies [11]. Based on our findings we recommend a minimum group size of at least 50 and a minimum of 50 groups to produce valid estimates for multilevel logistic regression models. We offer a caveat here such that the group size must be adjusted properly for low-prevalent outcomes; specifically the expected number of outcomes in each group should be greater than one. This caveat is offered as a caution to researchers using multilevel logistic regression in conjunction with small data sets; under these conditions researchers can expect to encounter convergence problems, large biases in their model estimates and inadequate statistical inference procedures. Our findings suggest that when choosing a sample size, researchers should base their decision on the level of bias that they consider acceptable for that particular study.

The main findings from this research can be summarized as follows: (i) convergence problems arise when prevalence is low, the number of groups is small, or the group size is small; (ii) the estimates of the fixed effect parameters are unbiased when the number of groups is relatively large (more than 50) and with moderate group size; (iii) when group size is small (e.g. 5) the estimates of the random slope and random intercept are severely overestimated, and; (iv) the standard errors of the variance component estimates are underestimated even with 100 groups and group size of 50.

## Competing interests

The author(s) declare that they have no competing interests.

## Authors' contributions

RM performed simulation studies and drafted the paper. RM, FIM and RHG conceptualized the research and revised the manuscript for intellectual content. All authors read and approved the final manuscript.

## Pre-publication history

The pre-publication history for this paper can be accessed here:


